# Supplementary material for: Regulatory Role of GgaR (YegW) for Glycogen Accumulation in Escherichia coli K-12
Source: Microorganisms. 2024 Jan 5;12(1):115. doi: 10.3390/microorganisms12010115 (PMC10819704; doi:10.3390/microorganisms12010115)
Supplement: Supplementary file 1 [file microorganisms-12-00115-s001.zip › microorganisms-2780719-supplementary.pdf]

microorganisms

MDPI

Table S1. Primers used in this study.

(A) For gel shift assay

Name	sequence
fbaB/yegT-F	CAAATGAAGCTCCGGCAGAGT
fbaB/yegT-R	CATGACAGGTAATGTAAGGTACGCG
fbaB/yegT1-F	CAATCATTACGCGGTCTACGTAGTC
fbaB/yegT1-R	TTTCTGATGAATCGAGCC
fbaB/yegT2-F	AATCATAATCCTGGTCGTTAC
fbaB/yegT2-R	GATAAACCATTCAACAAACATC
fbaB/yegT3-F	CTGTCATGAAGGAATTAAAAGA
fbaB/yegT3-R	GGCATATAGGTTAGCGAGTA
fbaB/yegT4-F	ATCACTGACCGCTTTTTCTC
fbaB/yegT4-R	CCAACTTCGGTCAGATAAC
fbaB/yegT5-F	AAATGGATATTAAAGTCATGCTC
fbaB/yegT5-R	ATAAAGAAGCCATAGCGGAT
fbaB/yegT6-F	AAGGTATTATTGCTTGGTCTG
fbaB/yegT6-R	TCATTTAACTTCCCCTTGTGTCAAC

(B) For Northern blot analysis

yegT-F	GTTGGCTCCATCACTGACCG
yegT-R	AACGCTAGTGGCATCGCAAA
yegV-F	GATTACACACGCTGCTGCCT
yegV-R	CGTCACGTCTTGCAGCTCTT
fbaB-F	TTGCGGTTGGTGCGACTATC
fbaB-R	GTCCTGCACGGCGTTAATCA

(C) For RT-qPCR

yegT-F	TTGCGCTGACTAACAGCATC
yegT-R	AAGAAACCACATGCCAGACC
yegV-F	AAGACGTGACGCCGTTTATC
yegV-R	TCTTGACGATTGAGCGACAC
fbaB-F	ACGCCGACAACCTTTTACAG
fbaB-R	TCATGTCCGGGGAGATAAAG

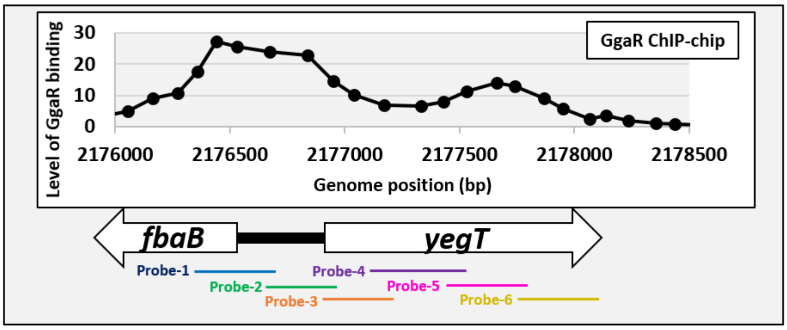

CAATCATTACGCGGTCTACGTAGTCATGTCCGGGGAGATAAAGCTGGTCAGAAGGAATTGTCATACAACGGTGCTGTAAAAGGTTGTCGGCGTCTTTGCC
Probe-1 FbaB start codon
AAGCAACTGCGCAATATCTGTCATTACTATGCTCCCGTAAATTCCGATTGGATATCGGCTATGGATTGTCCTGGCCCGCCTTTTGCGGGCAATCATAATC
Probe-2
CTGGTCGTTACGGGCGAGTTTTTCCATCTTTTGGATGTTTTTTCAGCGTTTTCTGTTGGCTCGATTCATCAGAAAAATGTTAGCGCGGTCAAGTTTTCA
YegT start codon
CCGCAAAGGTATTTAAAAGGTATTATTAAGTGGTATTGTCATCGCGTACCTTACATTACCTGTCATGAAGGAATTAAAAGATGAAAACAACAGCAAAGCT
Probe-3
GTCGTTCATGATGTTTGTTGAATGGTTTATCTGGGGCGCGTGGTTTGTGCCATTGTGGTTGTGGTTAAGTAAAAGCGGTTTTAGTGCCGGAGAAATTGGC
TGGTCGTATGCCTGTACCGCCATTGCGGCGATCCTGTCGCCAATTCTGGTTGGCTCCATCACTGACCGCTTTTTCTCGGCGCAAAAAGTGCTGGCGGTAT
Probe-4
TGATGTTCGCAGGCGCGCTGCTGATGTATTTCGCTGCGCAACAGACCACTTTTGCCGGGTTCTTCCCGTTACTGCTGGCCTACTCGCTAACCTATATGCC
GACCATTGCGCTGACTAACAGCATCGCTTTTGCCAACGTGCCGGATGTTGAGCGTGATTTCCCGCGCATTCGTGTGATGGGCACTATCGGCTGGATTGCC
TCCGGTCTGGCATGTGGTTTCTTGCCGCAAATACTGGGGTATGCCGATATCTCACCGACTAACATCCCGCTGCTGATTACCGCCGGAAGTTCTGCTCTGC
TCGGTGTGTTTGCGTTTTTCCTGCCCGACACGCCACCAAAAAGCACCGGCAAAATGGATATTAAAGTCATGCTCGGCCTGGATGCGCTGATCCTGCTGCG
Probe-5
CGATAAAAACTTCCTCGTCTTTTTCTTCTGTTCATTCCTGTTTGCGATGCCACTAGCGTTCTATTACATCTTTGCCAACGGTTATCTGACCGAAGTTGGC
ATGAAAAACGCCACCGGCTGGATGACGCTCGGCCAGTTCTCTGAAATCTTCTTTATGCTGGCATTGCCGTTTTTCACTAAACGCTTTGGTATCAAAAAGG
Probe-6
TATTATTGCTTGGTCTGGTCACCGCTGCGATCCGCTATGGCTTCTTTATTTACGGTAGTGCGGATGAATATTTCACCTACGCGTTACTGTTCCTCGGTAT
TTTGCTTCACGGCGTAAGTTACGATTTTTACTACGTTACCGCTTACATCTATGTCGATAAAAAAGCCCCCGTGCATATGCGTACCGCTGCGCAGGGGCTG
ATCACGCTCTGCTGCCAGGGCTTCGGCAGTTTGCTCGGCTATCGTCTTGGCGGTGTGATGATGGAAAAGATGTTCGCTTATCAGGAACCGGTAAACGGAC
TGACTTTCAACTGGTCCGGGATGTGGACTTTCGGCGCGGTGATGATTGCCATTATCGCCGTGCTGTTCATGATTTTTTTCCGCGAATCCGACAACGAAAT
YegT stop codon
TACGGCTATCAAGGTCGATGATCGCGATATTGCGTTGACACAAGGGGAAGTTAAAtga

Figure S1. Location of each probe in the *fbaB*/*yegT* region used to map the GgaR-binding site.

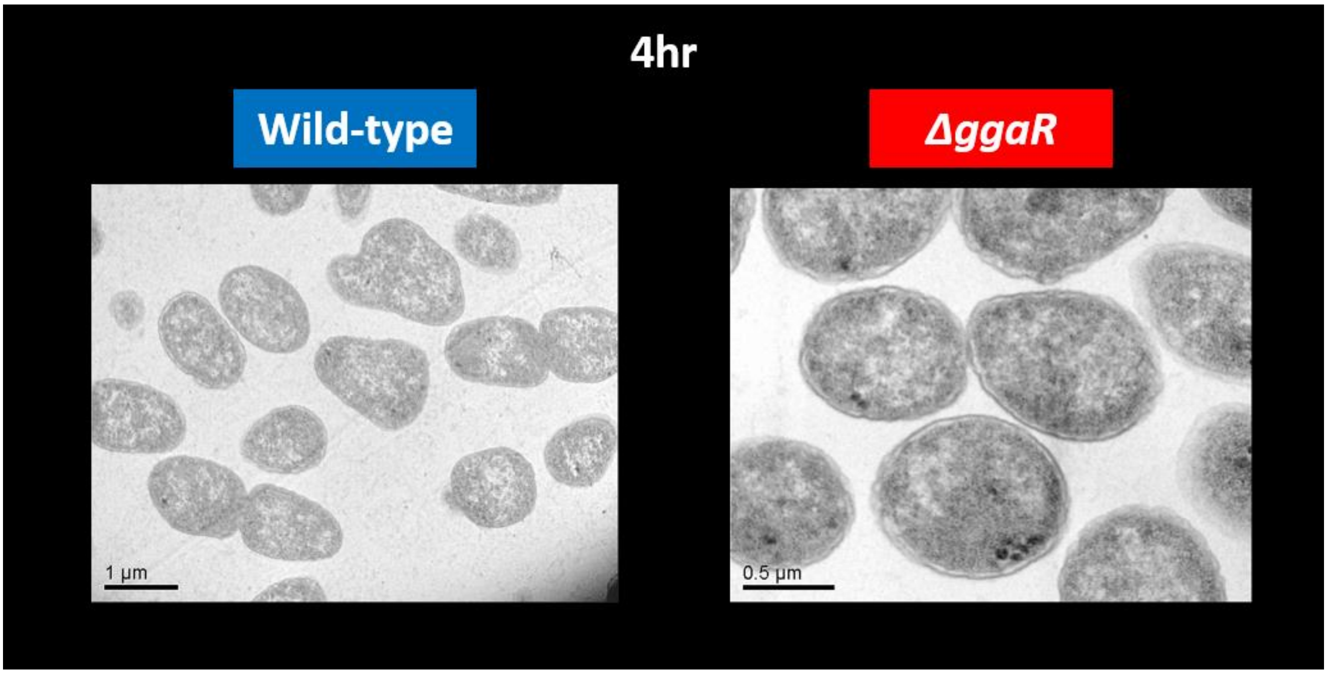

Figure S2. TEM analysis of glycogen granules 4 h after inoculation.
